# Zoonotic Disease Pathogens in Fish Used for Pedicure

**DOI:** 10.3201/eid1806.111782

**Published:** 2012-06

**Authors:** David W. Verner-Jeffreys, Craig Baker-Austin, Michelle J. Pond, Georgina S. E. Rimmer, Rose Kerr, David Stone, Rachael Griffin, Peter White, Nicholas Stinton, Kevin Denham, James Leigh, Nicola Jones, Matthew Longshaw, Stephen W. Feist

**Affiliations:** Centre for Environment, Fisheries & Aquaculture Science Weymouth Laboratory, Weymouth, UK (D.W. Verner-Jeffreys, C. Baker-Austin, M.J. Pond, G.S.E. Rimmer, R. Kerr, D. Stone, R. Griffin, P. White, N. Stinton, K. Denham, M. Longshaw, S.W. Feist);; University of Nottingham, Sutton Bonington, UK (J. Leigh);; Oxford Radcliffe University Hospitals, Headington, UK (N. Jones)

**Keywords:** pathogen, Vibrio vulnificus, Streptococcus agalactiae, bacteria, fish pedicure, doctor fish, Garra rufa, United Kingdom, spa, zoonoses

## Abstract

“Doctor” fish might not be such good doctors after all. These fish are used for the increasingly popular spa treatment called fish pedicures. During these sessions, spa patrons immerse their feet in water, allowing the live fish to feed on dead skin, mainly for cosmetic reasons. However, examinations of doctor fish destined for these spas found that they can carry harmful bacteria. Thus, although reports of human infection after fish pedicures are few, there may be some risks. Spa patrons who have underlying medical conditions (such as diabetes, immunosuppression, or even simple breaks in the skin) are already discouraged from taking such treatments. However, spas that offer fish pedicures should also consider using only disease-free fish reared in controlled facilities under high standards of husbandry and welfare.

**To the Editor:** Doctor fish (*Garra rufa*) are freshwater cyprinid fish that naturally inhabit river basins in central Eurasia. They are widely used in the health and beauty industries in foot spas for ichthyotherapy (Kangal fish therapy or doctor fish therapy) (Figure; Technical Appendix Figure 1) (*1*). During these sessions, patients immerse their feet or their entire bodies in the spas, allowing the fish to feed on dead skin for cosmetic reasons or for control of psoriasis, eczema, and other skin conditions.

**Figure:**
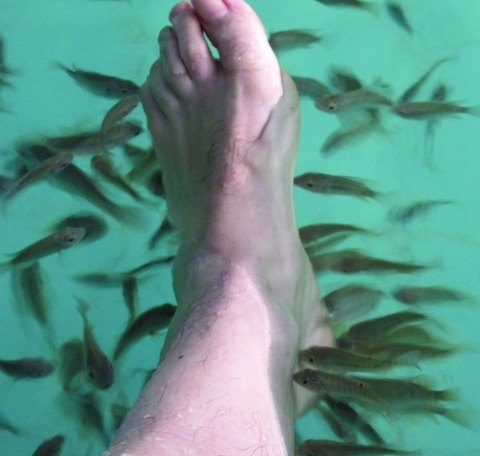
Doctor fish surrounding foot during ichthyotherapy.

A survey during the spring of 2011 identified 279 fish spas in the United Kingdom, and the number has probably increased since then (*1*). The Fish Health Inspectorate of the Centre for Environment, Fisheries & Aquaculture Science estimates that each week 15,000–20,000 *G. rufa* fish are imported from Indonesia and other countries in Asia into the United Kingdom through London Heathrow Airport (the main border inspection post for the import of live fish). However, ichthyotherapy has now reportedly been banned in several US states and Canada provinces because of sanitary concerns (*1*). In the United Kingdom, a limited number of infections after fish pedicures have been reported (*1*). Unfortunately, little is known about the types of bacteria and other potential pathogens that might be carried by these fish and the potential risks that they might pose to customers or to ornamental and native fish.

On April 12, 2011, the Fish Heath Inspectorate investigated a report of a disease outbreak among 6,000 *G. rufa* fish from Indonesia that had been supplied to UK pedicure spas. Affected fish showed clinical signs of exophthalmia and of hemorrhage around the gills, mouth, and abdomen. More than 95% of the fish died before the remaining fish were euthanized. Histopathologic examinations identified systemic bacterial infections with small gram-positive cocci, mostly in the kidneys, spleen, and liver. Bacterial isolates cultured from affected fish were identified as *Streptococcus agalactiae* (group B *Streptococcus*) according to a combination of biochemical test results (API Strep; bioMérieux, Marcy l’Étoile, France), Lancefield grouping with serotype B (Oxoid Limited, Basingstoke, UK), and molecular (partial 16S rRNA gene sequencing) testing methods.

Multilocus sequence typing of a representative isolate (11013; Technical Appendix Table) (*2*) indicated that it was a sequence type (ST) 261 *S.* *agalactiae* strain (http://pubmlst.org/sagalactiae). This same ST261 profile was first identified in an isolate (ATCC 51487) from a diseased tilapia in Israel (*3*). The clinical appearance of the disease and the diagnostic results suggested that *S. agalactiae* was the causative agent of the fish illness and deaths.

To determine whether *S. agalactiae* and other bacterial pathogens might be carried more widely by these fish, from May 5, 2011, through June 30, 2011, the Fish Health Inspectorate of the Centre for Environment, Fisheries & Aquaculture Science visited Heathrow Airport 5 times to intercept and sample consignments of *G. rufa* from Indonesia. A taxonomically diverse range of bacteria were identified (Technical Appendix Table, Figure 2), including a variety of human pathogens capable of causing invasive soft tissue infections. These pathogens included *Aeromonas* spp (*4*), potentially pathogenic clinical-type *Vibrio vulnificus* isolates (Technical Appendix Figure 2) (*5*), non–serotype O1 or O139 cholera toxin–negative *V. cholerae* isolates (Technical Appendix Figure 2) (*6*), *Mycobacteria* (*7*), and *S. agalactiae* (*3**,**8*). Isolates were resistant to a variety of antimicrobial drugs, including tetracyclines, fluoroquinolones, and aminoglycosides (Technical Appendix Table). Other studies have also reported high levels of multidrug resistance in bacteria associated with imported ornamental fish (*9*).

Water is a well-recognized source of bacterial skin infections in humans. *V. vulnificus* can cause wound infections and primary septicemia, resulting in high mortality rates, especially among persons with predisposing risk conditions (e.g., liver disease, diabetes, or impaired immune function) (*5*). *S. agalactiae* is a common cause of skin and soft tissue infections, especially in older adults and those with chronic diseases such as diabetes mellitus (*8*). Although *S. agalactiae* ST261 is not considered to be one of the genotypes typically associated with invasive disease in humans (*3*), a fish-adapted strain could eventually take advantage of the opportunity afforded by repeated exposure and thereby also affect humans. Additionally, *Mycobacteria* spp. can occasionally cause disease in humans through contact with fish (*M. marinum*), and pedicure treatments have previously been associated with *M. fortuitum* infections (*10*).

Recently, the risks associated with exposure to *G. rufa* fish were reported to be low (*1*). To date, there are only a limited number of reports of patients who might have been infected by this exposure route (*1*). However, our study raises some concerns over the extent that these fish, or their transport water, might harbor potential zoonotic disease pathogens of clinical relevance. In particular, patients with underlying conditions (such as diabetes mellitus or immunosuppression) should be discouraged from undertaking such treatments, especially if they have obvious breaks in the skin or abrasions. This risk can probably be reduced by use of certified disease-free fish reared in controlled facilities under high standards of husbandry and welfare.

## Supplementary Material

Technical Appendix Identities, source, and antimicrobial drug resistance profiles of bacterial isolates from *Garra rufa* fish shipments from Indonesia, doctor fish surrounding foot during ichthyotherapy, and agarose gel electrophoresis image of *Vibrio* spp.–specific PCR analyses specific for the 310-bp pRVC gene fragment in *Vibrio cholerae* and the 519-bp vvHA gene fragment in *V. vulnificus*.
