# Supplementary material for: Zoonotic Disease Pathogens in Fish Used for Pedicure
Source: Emerg Infect Dis. 2012 Jun;18(6):1006–8. doi: 10.3201/eid1806.111782 (PMC3358165; doi:10.3201/eid1806.111782)
Supplement: Technical Appendix — Identities, source, and antimicrobial drug resistance profiles of bacterial isolates from Garra rufa fish shipments from Indonesia, doctor fish surrounding foot during ichthyotherapy, and agarose gel electrophoresis image of Vibrio spp.–specific PCR analyses specific for the 310-bp pRVC gene fragment in Vibrio cholerae and the 519-bp vvHA gene fragment in V. vulnificus. [file 11-1782-Techapp_3p.pdf]

Article DOI: http://dx.doi.org/10.3201/eid1806.111782

Zoonotic Disease Pathogens in Fish Used for Pedicure

Technical Appendix

Technical Appendix Table. Identities, source, and antimicrobial drug resistance profiles of bacterial isolates from *Garra rufa* fish shipments from Indonesia, sampled at Heathrow Airport, London, UK, May 5, 2011–June 30, 2011. Antimicrobial drug susceptibility was determined for the isolates against 28 antimicrobial drugs from 13 structural classes, using broth-microdilution assays (Sensititer AVIAN and GN2 panels; Trek Diagnostic Systems, East Grinstead, UK). Testing followed guidelines from the Clinical and Laboratory Standards Institute, as described (*1*).

Isolate	Identity‡ (closest RDP sequence; S_ab§)	Date, 2011	Resistance phenotype¶
11016	*Aeromonas veronii (*X60414;1.00¶	May 5	(Enr),Otc, Tet, Amx, Pen, Fep, (Ami),(Str), (Spe), Cip, Gat
11040	*Aeromonas* spp. (X74674; 1.00)¶	May 5	Enr, Tio, Otc, Tet, Amx, Pen, (Ffn),Str, Spe, Faz, (Fur), Cip, Gat, Tob
11021	*A. veronii*† (X60414; 0.99 ¶	May 9	Enr, Otc, Tet,Pen,(Str), Tob
11052	*Enterobacteria kobei* (AJ508301; 0.951)	Jun 24	Amx, Pen, Faz,
11053	*E. kobei* (AJ508301; 0.951)	Jun 24	Amx, Pen, Faz,
11038	*Citrobacter freundi* (AJ233408; 0.977)	Jun 8	Otc, Tet,Pen,(Nit),
11023	*Mycobacterium senegelense* (AY684063;100%)§	May 9	Not done
11024	*M. senegelense* (AY684063;100%)§	May 9	Not done
11055	*Aeromonas* spp. (X60408; 0.946)¶	Jun 30	(Otc), (Tet), Pen, (Str),
11056	*Aeromonas* spp. (X60408; 0.946 ¶	Jun 30	Otc, (Tet),Gen
11022	*Shewanella* spp.(AB205566; 0.934)	May 9	Enr,Otc, Tet,Faz, (Mero), Gen
11037	*Shewanella* spp.(AB205566; 1.000)	Jun 8	Not Done (isolate lost on subculture)
11013	*Streptococcus agalactiae* (AB112407; 1.00)	Apr 12	Str, Gen, †
11041	*Acinetobacter* spp.(AJ293694; 0.905)	Jun 8	Enr, Otc, Tet, (Spe), (Ffn),
11050	*Chryseobacterium* spp.(EF685359; 0.858)	Jun 24	Enr, Otc,Tet,(Amx), Pen,Str, (Ami), Sxt, Azt, Faz, Tans,Tur, Cip, Gat, Tob, Pod
11054	*Chryseobacterium* spp. (AM232813; 0.907)	Jun 24	Enr, Tio,Otc, Tet, Amx, (Spe), Str, Ami, Azt, Faz, Tans, Taz, Fur, Cip, (Gat), Gen, Fox, (Tim), Tob, Pod
11011	*Vibrio vulnificus* (X74727; 1.00)	Apr 12	(Str), (Faz)
11039	*V. cholerae* (NAG) (X76337;0.985)	Jun 8	(Otc), (Tet), Amx,
11051	*V. vulnificus* (X74727; 1.00)	Jun 24	(Spe), Str
11012	*V. cholerae* (NAG) (X76337;0.985)	Apr 12	Enr, Otc, Tet, Cip (Gat)
11036	*V. mimicus* (X74713; 0.975)	May 18	Pen

*Isolates 11052 and 11053 were from a UK retail outlet; all others were from consignments originating from Indonesia. Isolates represent the range of dominant colony morphotypes observed, recovered from imported G. rufa fish. Unless otherwise indicated, all tests reported were performed at 37 ± 2°C and results read after between 24–72 h incubation.

Ami, amikacin; Amx, amoxicillin; Axo, ceftriaxone; Cpr, Ciprofloxacin, Eno, enrofloxacin; Faz, cefazolin; Ffl, florfenicol; Fox, cefoxitin; Fur, cephalothin; Gat, gatifloxacin; Gen, gentamicin; Imi, imipenem; Neo, neomycin; Nit, nitrofurantoin; Pod, cefpodoxime; Str, streptomycin; Pip, piperacillin; Spe, spectinomycin; Str, streptomycin; Sxt, sulfamethoxazole/trimethoprim; Tans, cefotetan Na; Taz, ceftazidime, Tio, ceftiofur; Tob, tobramycin. The range of concentrations of antimicrobial drugs and, in the absence of published break points for the gram-negative organisms tested, the epidemiologic cut-off tolerance values used, were as described (*1*). Codes in parentheses indicate isolate was of intermediate tolerance to indicated antimicrobial drug.

†Tested at 22°C because growth on Mueller Hinton media at 37°C was poor.

‡All bacterial isolates were identified on the basis of phenotypic testing criteria (Gram stain, cytochrome oxidase and catalase activity and motility), with gram-negative isolates identified further using API 20NE and API 20E (Biomérieux) miniaturized biochemical test systems and partial 16S rRNA gene sequencing. Partial 16S rRNA sequences were aligted to the closest relative using the Ribosomal Database Project SeqMatch tool (http://rdp.cme.msu.edu/seqmatch/). Except for isolates 11023 and 11024, closest partial 16S rRNA gene sequence derived from a Type, or otherwise well-characterized strain, displayed and RDP SeqMatch score (S_ab), with 1.0 representing total sequence identity).

§Species assignment of *Mycobacterium* sp. isolates was based on both partial 16S rRNA and partial RNA polymerase β gene sequencing (*2*)). Closest RNA polymerase β gene sequence relative obtained by using BLAST (http://blast.ncbi.nlm.nih.gov/Blast.cgi) is displayed.

¶Partial 16S rRNA gene sequencing, species complex assignment of *Aeromonas* spp. isolates were also confirmed based on direct BLAST comparison of partial gyrA DNA gene-sequencing (*3*). Partial 16S and *gyr*A sequences that together shared <95% similarity with a well-characterized strain the organism are listed as an *Aeromonas* spp. only.

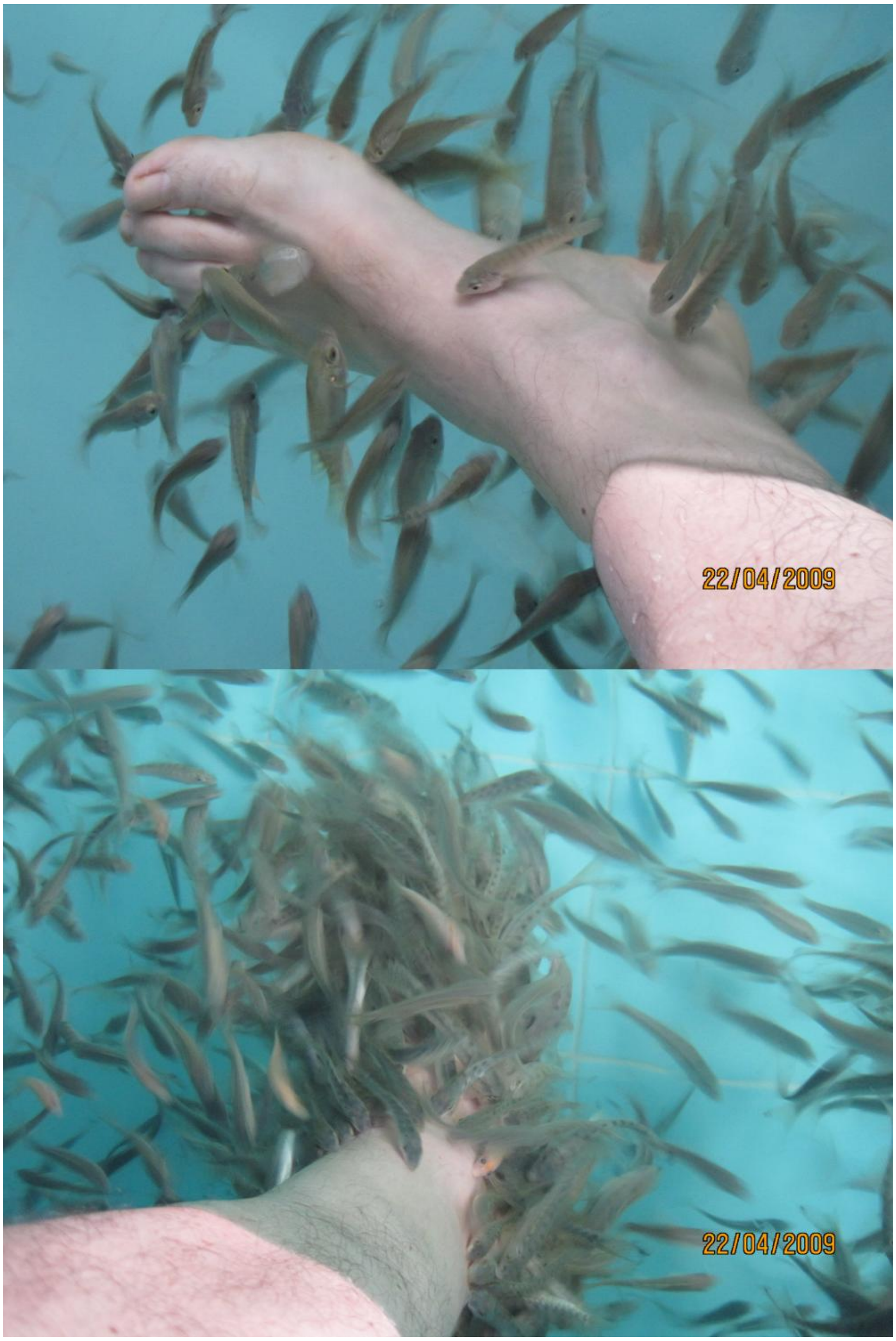

Technical Appendix Figure 1. Doctor fish surrounding foot during ichthyotherapy.

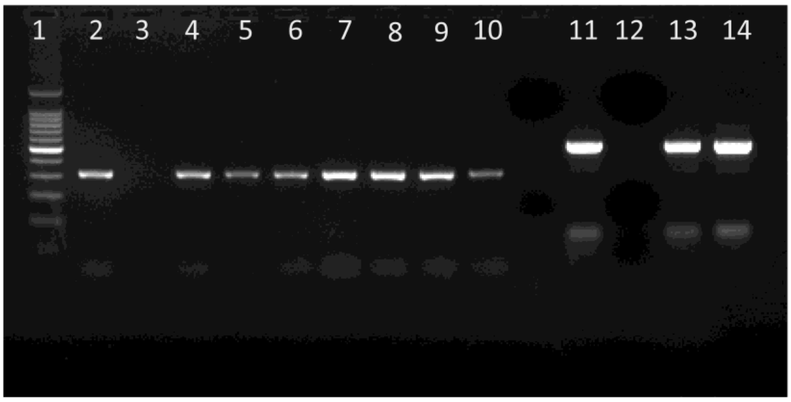

Technical Appendix Figure 2. Agarose gel electrophoresis image of *Vibrio* spp.–specific PCR analyses specific for the 310-bp *pRVC* gene fragment in *V. cholerae* (*4*) and the 519-bp *vvHA* gene fragment in *V. vulnificus* (*5*). Lane 1, 100-bp DNA ladder (Invitrogen, Carlsbad, CA, USA). Lane 2, positive-control material (*V. cholerae* National Collection of Type Cultures [NCTC] strain 8042). Lane 3, negative control (water), Lanes 4–10, 7 presumptive *V. cholerae* strains isolated on thiosulfate–citrate–bile salts sucrose agar plates and confirmed by PCR. Lane 11, *V. vulnificus*–positive control material (NCTC11067). Lane 12, negative control (water). Lanes 13 and 14, *V. vulnificus* strains 20 and 129.

References

1. Verner-Jeffreys DW, Welch TJ, Schwarz T, Pond MJ, Woodward MJ, Haig SJ, et al. High prevalence of multidrug-tolerant bacteria and associated antimicrobial resistance genes isolated from ornamental fish and their carriage water. PLoS ONE. 2009;4:e8388. PubMed http://dx.doi.org/10.1371/journal.pone.0008388

2. Chun J, Huq A, Colwell RR. Analysis of 16S–23S rRNA intergenic spacer regions of *Vibrio cholerae* and *Vibrio mimicus*. Appl Environ Microbiol. 1999;65:2202–8. PubMed

3. Hor LI, Gao CT, Wan L. Isolation and characterization of *Vibrio vulnificus* inhabiting the marine environment of the southwestern area of Taiwan. J Biomed Sci. 1995;2:384–9. PubMed http://dx.doi.org/10.1007/BF02255226

4. Adékambi T, Colson P, Drancourt M. rpob-based identification of nonpigmented and late-pigmenting rapidly growing mycobacteria. J Clin Microbiol. 2003;41:5699–708. PubMed http://dx.doi.org/10.1128/JCM.41.12.5699-5708.2003

5. Goñi-Urriza M, Arpin C, Capdepuy M, Dubois V, Caumette P, Quentin C. Type II topoisomerase quinolone resistance-determining regions of *Aeromonas caviae*, *A. hydrophila*, and *A. sobria* complexes and mutations associated with quinolone resistance. Antimicrob Agents Chemother. 2002;46:350–9. PubMed http://dx.doi.org/10.1128/AAC.46.2.350-359.2002
